# Do the Approved Societies Seek to Control the Doctors?

**Published:** 1922-08

**Authors:** R. W. Harris

**Affiliations:** (formerly Assistant Secretary, responsible for the administration of National Health Insurance Medical Benefits, Ministry of Health)


### DO THE APPROVED SOCIETIES SEEK TO CONTROL THE DOCTORS?

**By R. W. HARRIS (formerly Assistant Secretary, responsible for the administration of National Health Insurance Medical Benefits, Ministry of Health).**

SOME questions are asked in headlines in order that the subject may be explored. I have asked this question in order to answer it. The answer is in the negative. So much has been said in and out of Parliament during the last few weeks on the question of how the cost of Medical Benefit has been, is being, and is going to be met, that readers might well be pardoned for getting their ideas on the subject a little bit tangled. And through the maze many insurance doctors try to peer, and they ask, uneasily, whether there is not some subtle move on foot on the part of the Approved Societies to get control of the panel system and dictate terms of service to the doctors. In the circumstances, it may not be amiss to state shortly what has happened.

In 1911, the financial scheme of the National Insurance Bill included a sum of 6s. per head of the insured population for Medical Benefit (doctors and chemists). This was to come, as to $\frac{7}{9}$ from the contributions of the employed person and his employer, and $\frac{2}{9}$ from the Exchequer; out of every 9d., 4d. was to be found by the employed insured person, 3d. by his employer, 2d. by the State. Hence "ninepence for fourpence." Medical Benefit was not forthcoming at the price, and the 6s. became 9s. Of this 6d. was taken from the Sanatorium Benefit Funds (for domiciliary treatment) and a new 2s. 6d. was found by the taxpayer. In this way contributions found $\frac{7}{9}$ of 6s. 6d., and the Exchequer found 2s. 6d. plus $\frac{2}{9}$ of 6s. 6d.; the resulting amounts show that the Exchequer $\frac{2}{9}$ (the fraction paid for all other benefits) had already become $\frac{4}{9}$, before the medical provisions of the Act had started. When the cost of living went up in the closing days of the War, additional amounts (by way of temporary war bonus) were found by the Exchequer.

Then came the demand for a definite increase in the remuneration of doctors and chemists, with arbitration in the case of the former. The total cost increased from the beginning of 1920 to 13s. 9d. The weekly insurance contributions, *partly as a direct result of this increase,* were increased by the National Insurance Act of 1920, and by the same Act the cost of medical benefit was required to be met out of insurance funds to the extent of 9s. 6d., $\frac{7}{9}$ of this amount coming from contributions. So that the Exchequer continued to find the balance of the 13s. 9d. (4s. 3d.) as well as $\frac{2}{9}$ of 9s. 6d.—a total of nearly 6s. 6d. out of 13s. 9d., or rather more than the $\frac{4}{9}$ which the Exchequer had all along been paying, and more than double the share ($\frac{2}{9}$) which had been originally provided for in the Act of 1911.

A reduction in the remuneration of the doctors and the chemists this year has been effected, the relief falling entirely to the Exchequer, but the Geddes Committee recommended that the Exchequer should be further relieved and should get back to the $\frac{2}{9}$ basis laid down by the Act of 1911. The Government adopted this recommendation. The total cost of medical benefit—at any rate up to the end of 1923—being now about 12s. a year, and 9s. 6d. only being provided for in the Act of 1920, the question arose whether Parliament should be asked to increase the weekly insurance contribution of the employer or the employed person, or both. The Consultative Council, which embodies Approved Society experience, advised that this should not be done at the present time. They recommended that up to the end of 1923 the money required over and above the statutory amount of 9s. 6d. should be found by drawing on the accumulated surpluses in the benefit funds of Approved Societies. The Government Actuary gave this proposal his qualified blessing, the qualification being that the benefit funds must not be expected to stand any further strain.

It has nowhere been stated that the Consultative Council conditioned this proposal by any request that Approved Societies should in future be parties to the settlement of the terms of service of doctors or chemists, nor indeed were they in a position to make such a condition. The Approved Societies have come to the temporary assistance of the Exchequer for the express purpose of avoiding the imposition of an additional insurance contribution at the present time. The offer having been accepted, a Bill has been laid before Parliament to give effect to the proposal. It has not had altogether a smooth passage. With the suggestions made in debate that the Consultative Council does not truly express Societies' opinion, with the defeat of the Government in Committee on the question whether the money should come from the Central Fund rather than the Benefit Funds, and with the subsequent modification of the Bill whereby the Central Fund contributes one-third of the amount, this article is not concerned, except, in passing, to say, on the last-named point, that the effect on Societies of taking money from the Central Fund (available, on certain conditions, for future deficiencies) is more remote and indirect than the taking of money from existing ascertained surpluses in the Benefit Funds.

"Having regard to the provision in this Bill for contributions in relief of State expenditure from the surplus funds of Approved Societies," said Sir Alfred Mond on the second reading of the Bill, "I think that it is no more than fair and reasonable that I shall give the Societies an opportunity of expressing their views before any new terms of service are made with the panel practitioners. But the responsibility for accepting these contracts rests and will continue to rest with the Minister of Health." And again, "There is no word in this Bill which modifies the existing statutory provision for the control of medical benefit."

Nothing could be clearer. The Societies are not vested by Parliament with the responsibility for administering medical benefit. They can, of course, as voicing the views of insured persons, promise, if more than $x$ is taken from the contributions of employed persons, to make trouble in Parliament and in the country. It is a power which employers also possess and which the taxpayer shares. But the responsibility to see that the service is forthcoming is laid upon the Minister, and he must therefore, negotiate the terms on which that service can be secured.

It is because of the express statement of the Minister in the House, and in the light of the history of medical benefit administration, that I have ventured to make the assertion at the beginning of this article. I believe that the Societies want to see the doctors well paid, and above all, that their insured members are well treated. And on the last point, it may be observed that insured persons' representatives have a three-fifths (*i.e.*, a majority) representation on every Insurance Committee in the country.

